# NUF2 overexpression contributes to epithelial ovarian cancer progression *via* ERBB3-mediated PI3K-AKT and MAPK signaling axes

**DOI:** 10.3389/fonc.2022.1057198

**Published:** 2022-12-21

**Authors:** Ruobing Leng, Yunfang Meng, Xiaomei Sun, Yingzi Zhao

**Affiliations:** ^1^ Department of Gynecology, Shandong Provincial Hospital Affiliated to Shandong First Medical University, Jinan, Shandong, China; ^2^ Department of Dermatology, Shandong Provincial Hospital Affiliated to Shandong First Medical University, Jinan, Shandong, China; ^3^ Department of Obstetrics, Shandong Provincial Hospital Affiliated to Shandong First Medical University, Jinan, Shandong, China

**Keywords:** NUF2, ERBB3, epithelial ovarian cancer, AKT, MAPK

## Abstract

**Introduction:**

NDC80 kinetochore complex component (NUF2) is upregulated and plays an important role in various human cancers. However, the function and mechanism of NUF2 in epithelial ovarian cancer (EOC) remain unclear.

**Methods:**

NUF2 expression was detected in EOC tissues and cell lines. The effects of NUF2 downregulation on cell proliferation, migration and invasion in EOC were analyzed by CCK-8 and Transwell assays. Meanwhile, the effect of NUF2 downregulation on tumor growth in vivo was determined by xenograft tumor models. The mechanisms by which NUF2 regulates EOC progression were detected by RNA sequencing and a series of in vitro assays.

**Results:**

We showed that NUF2 was significantly upregulated in EOC tissues and cell lines, and high NUF2 expression was associated with FIGO stage, pathological grade and poor EOC prognosis. NUF2 downregulation decreased cell proliferation, migration, invasion and tumor growth in nude mice. RNA sequencing studies showed that NUF2 knockdown inhibited several genes enriched in the phosphatidylinositol-4,5-bisphosphate 3-kinase (PI3K)-AKT serine/threonine kinase (AKT) and mitogen-activated protein kinase (MAPK) signaling pathways. Erb-B2 receptor tyrosine kinase 3 (ERBB3) was the key factor involved in both of the above pathways. We found that ERBB3 silencing could inhibit EOC progression and repress activation of the PI3K-AKT and MAPK signaling pathways. Furthermore, the exogenous overexpression of ERBB3 partially reversed the inhibitory effects on EOC progression induced by NUF2 downregulation, while LY294002 and PD98059 partially reversed the effects of ERBB3 upregulation.

**Conclusion:**

These results showed that NUF2 promotes EOC progression through ERBB3-induced activation of the PI3K-AKT and MAPK signaling axes. These findings suggest that NUF2 might be a potential therapeutic target for EOC.

## 1 Introduction

Ovarian cancer is the fifth most lethal malignancy in women, and epithelial ovarian cancer (EOC) is the most common histological type (1). Due to the absence of specific symptoms and diagnostic biomarkers, greater than 70% of EOC patients are diagnosed with clinical stage Federation International of Gynecology and Obstetrics (FIGO) III or IV, which has a five-year survival rate of only 20% to 30% (1). Several studies showed that a multidisciplinary approach for the treatment of ovarian cancer has significantly improved the quality of life and prognosis of patients and is now a well-established part of clinical care (2–5). A multidisciplinary team is able to face clinical, molecular, pathological and psychological issues of patients with ovarian cancer, ensuring a high standard of care supporting the process of personalized medicine. Although the multidisciplinary approach has improved the quality of life of patients, it is still urgent to continuously improve the molecular, biological and therapeutic knowledge in the field of ovarian cancer care. Thus, it is crucial to identify novel molecular biomarkers and therapeutic targets for diagnosis and the in-depth understanding of the molecular pathogenesis of EOC.

NDC80 kinetochore complex component (NUF2), also named cell division associated 1 (CDCA1), was first reported as a centromere protein and is a key element of the Ndc80 kinetochore complex (6). Further studies revealed that NUF2 binds to centromere protein E (CENPE) and is required for stable spindle kinetochore-microtubule attachment (7). Evidence has shown that NUF2 is overexpressed in a series of human cancers and is significantly associated with poor prognosis (8–12). For example, NUF2 mRNA is significantly upregulated in breast cancer, and upregulated NUF2 is significantly associated with malignant features and poor prognosis (10). In kidney renal clear cell carcinoma (KIRC), NUF2 mRNA and protein are also significantly upregulated, and NUF2 mRNA is an independent prognostic risk factor for KIRC patients (11). Moreover, NUF2 contributes to the malignant progression of tumor, including colorectal cancer, gastric cancer, pancreatic cancer, breast cancer, and renal clear cell carcinoma (9, 13–16). For instance, NUF2 knockdown inhibited cell proliferation and colony formation and induced apoptosis in breast cancer (9). Likewise, NUF2 knockdown by siRNA significantly inhibited cell proliferation and induced apoptosis in colorectal cancer and gastric cancer cells (15). These results suggest that NUF2 may be a good candidate for molecular targeted therapy as well as diagnosis in some cancers. Interestingly, NUF2 has been reported to be upregulated in ovarian cancer, and NUF2 knockdown by small interfering RNA (siRNA) inhibited cell viability and induced apoptosis (17). However, the role and precise mechanism of NUF2 in ovarian cancer progression remain unclear.

In the present study, we found that NUF2 is highly expressed in human EOC specimens compared with normal ovarian epithelial tissues. The high expression of NUF2 was associated with poor EOC prognosis. NUF2 knockdown inhibited cell proliferation, migration and invasion through the Erb-B2 receptor tyrosine kinase 3 (ERBB3)-mediated phosphatidylinositol-4,5-bisphosphate 3-kinase (PI3K)-AKT serine/threonine kinase (AKT) and mitogen-activated protein kinase (MAPK) signaling axes.

## 2 Material and methods

### 2.1 Patients and specimens

In our study, a total of 109 paraffin-embedded tissue samples, including 89 EOC tissues and 20 normal ovarian epithelial tissues, were retrieved from the archives of the Department of Pathology, Shandong Provincial Hospital Affiliated to Shandong First Medical University, China, between May 2010 and August 2015. None of the patients were treated with chemotherapy or radiotherapy before they underwent surgery. The specimens were used with the written informed consent from the patients and the approval of the Ethics Committee of Shandong Provincial Hospital Affiliated to Shandong First Medical University (Approval No. 2021-774). The study was performed in accordance with the ethical standards as laid down in the 1964 Declaration of Helsinki and its later amendments or comparable ethical standards. Follow-up was performed monthly for the first year, then quarterly until 2 years, every 6 months until 3 years, and once 3 year thereafter. Medical examination and telephonic interviews were performed for follow-up and those who survived beyond March 20, 2018 were recorded as censored data. Patients were considered lost to follow-up if no further medical records and no record of death existed. In these cases, patients were censored at the time of their last medical encounter. Patients were excluded if they were lost to follow-up within six months.

### 2.2 Immunohistochemical staining

The paraffin-embedded blocks were sectioned at a thickness of 4 µm. After deparaffinization, rehydration, antigen retrieval and blocking of endogenous peroxidases, the sections were washed with PBS and incubated in normal goat serum at 37°C for 30 min. The tissues were subsequently incubated with anti-NUF2 antibody (1:100; ab230313, Abcam, Waltham, MA, USA) overnight at 4 °C. After washing with PBS, tissues were incubated with peroxidase-labeled secondary antibody at 37°C for 1 hour. The sections were visualized after DAB staining and counterstaining with hematoxylin.

The NUF2 staining score was determined as previously described (18). The staining intensity was scored as follows: 0, no staining or only weak staining; 1, moderate staining; and 2, strong staining. The positive proportion of stained tumor cells was scored as follows: 0, ≤ 5% positive cells; 1, 6–50% positive cells; 2, ≥ 51% positive cells. The NUF2 staining score was the sum of the staining intensity score and the positive staining cell rate score: 0–2, low expression; 3–4, high expression.

### 2.3 Cell culture

The CAOV3, OVCAR3 and SKOV3 cell lines were purchased from the Cell Bank of the Type Culture Collection of the Chinese Academy of Science (Shanghai, China), the A2780 cell line was obtained from Huiying Biotechnology Co., Ltd. (Shanghai, China), and the KGN cell line was obtained from Procell Life Science & Technology Co., Ltd. (Wuhan, China). The CAOV3, A2780 and KGN cell lines were cultured in DMEM (Thermo Fisher Scientific Inc., Waltham, MA, USA) supplemented with 10% (v/v) fetal bovine serum (FBS) (Thermo Fisher Scientific Inc., Waltham, MA, USA), OVCAR3 cells were maintained in RPMI-1640 medium (Thermo Fisher Scientific Inc., Waltham, MA, USA) supplemented with 10% (v/v) FBS, and SKOV3 cells were cultured in McCoy’s 5A medium (Thermo Fisher Scientific Inc., Waltham, MA, USA) supplemented with 10% (v/v) FBS, all in a humidified atmosphere of 5% CO_2_ at 37°C.

### 2.4 Plasmid construction and transfection

NUF2 and ERBB3 small hairpin RNAs (shRNAs) were synthesized and cloned into the phU6-shRNA-CMV-puromycin vector by Sesh-Biotech (Shanghai, China). The sequences of NUF2 shRNA (shNUF2) and ERBB3 shRNA (shERBB3) were shown in **Supplemental Table S1**. To overexpress ERBB3, ERBB3 cDNA was amplified and subcloned into pcDNA3.1 with hygromycin (Sino Biological, Inc., Shanghai, China).

The shNUF2, shERBB3 and ERBB3 overexpression (ERBB3-ov) plasmids were transfected into A2780 and OVCAR3 cells (3x10^5^ cells per well in 6-well plates) using Lipofectamine 2000 (Thermo Fisher Scientific Inc., Waltham, MA, USA) according to the manufacturer’s protocols.

### 2.5 RNA sequencing

Total RNA of OVCAR3 cells after transfection with shNC and shNUF2 was extracted in accordance with the manual of TRIzol™ reagent (Thermo Fisher Scientific Inc., Waltham, MA, USA). RNA integrity was analyzed *via* agarose gel electrophoresis. Library preparation and transcriptome sequencing on the Illumina NovaSeq 6000 (Illumina, San Diego, CA, USA) were carried out at Personalbio Technology Co., Ltd. (Shanghai, China). Fold change (FC) was used to describe the differentially expressed genes (DEGs). DEGs with FC values greater than 1 or less than -1 and a *P* value less than 0.05 were considered significant. Gene ontology (GO) term and kyoto encyclopedia of genes and genomes (KEGG) pathway enrichment analyses were carried out Personalbio Technology Co., Ltd. (Shanghai, China). The KEGG pathway maps were obtained from the KEGG database (http://www.kegg.jp/).

### 2.6 Real-time quantitative PCR

The total RNA of A2780 and OVCAR3 cells after transfection with shNC and shNUF2 was extracted with the TRIzol™ reagent (Thermo Fisher Scientific Inc., Waltham, MA, USA) according to the manufacturer’s instructions, and M-MLV reverse transcriptase (Promega Corporation, Madison, WI, USA) was used to synthesize cDNA. ERBB3 mRNA expression was analyzed using SYBR Master Mix (Takara, Dalian, China). The thermocycling conditions were as follows: Pre-denaturation at 95˚C for 15 sec, followed by 40 cycles of denaturation at 95˚C for 5 sec, annealing at 60˚C for 30 sec and extension at 60˚C for 30 sec. Relative quantification of ERBB3 mRNA was determined using the 2^−ΔΔCt^ method after normalization to the GAPDH. The PCR primers used in this study were shown in **Supplemental Table S2**.

### 2.7 Western blot

Cells were lysed using RIPA lysis buffer (Beyotime, Shanghai, China) supplemented with 1 mM phenylmethanesulfonyl fluoride (PMSF). After centrifugation at 14,000 rpm for 10 min at 4°C, a BCA protein assay kit (Beyotime, Shanghai, China) was used to determine the protein concentrations. Equal amounts of total proteins from each sample were loaded onto a 12.5% SDS-PAGE gel and transferred to PVDF membranes. The membranes were blocked and then incubated with primary antibodies at 4°C overnight. The primary antibodies used in this study were as follows: anti-NUF2 (1:800; 15731-1-AP, Proteintech Group, Inc, Wuhan, China), anti-ERBB3 (1:500; 10369-1-AP, Proteintech Group, Inc, Wuhan, China), anti-AKT (1:2,000; 10176-2-AP, Proteintech Group, Inc, Wuhan, China), anti-p-AKT (Ser473) (1:3,000; 28731-1-AP, Proteintech Group, Inc, Wuhan, China), anti-ERK1/2 (1:10,000; Abcam, Waltham, MA, USA), anti-p-ERK1/2 (Thr202/Tyr204) (1:1,000; #4695, Cell Signaling Technology, Boston, MA, USA) and anti-GAPDH (1:10,000; 10494-1-AP, Proteintech Group, Inc, Wuhan, China). The membranes were washed and incubated with the appropriate secondary antibodies. The target proteins bands on the membranes were detected using an ECL Western blotting Detection Kit (Beyotime, Shanghai, China). The gray values of the protein bands were analyzed using Quantity One software and normalized to GAPDH.

### 2.8 Cell viability assay

Cell viability was determined by a CCK-8 kit (Beyotime, Shanghai, China). After treatment, cells were seeded in 96-well plates (1×10^4^ cells per well) and maintained for 0, 24, 48, 72 and 96 h. 10 μl of CCK-8 solution were added to each well of the plate. After incubation for 2 h, the absorbance at 450 nm was determined using a microplate reader (Bio-Rad, Hercules, CA, USA).

### 2.9 Transwell assay

The cell migration ability was determined using Transwell chambers (BD Biosciences, San Jose, CA, USA) with a pore size of 8 μm, and the cell invasion ability was analyzed using Matrigel-coated Transwell chambers (BD Biosciences, San Jose, CA, USA) with a pore size of 8 μm. After treatment, A2780 and OVCAR3 cells were seeded onto the upper chambers at a final concentration of 4×10^4^ cells/well and cultured in 100 μl of serum-free medium. Complete medium was added to the lower chamber. After incubation for 48 h, the cells in the upper chamber were removed with a cotton swab. Cells on the lower surface of the membrane were stained with crystal violet, and the number of cells was counted in five random fields under a light microscope.

### 2.10 Nude mouse model

Four-week-old BALB/c male nude mice were obtained from the Animal Center of the Chinese Academy of Science (Shanghai, China). Stable NUF2-silenced OVCAR3 cells (shNUF2, 4×10^6^ cells in 100 μl of sterilized PBS) and stable negative control OVCAR3 cells (shNC, 4×10^6^ cells in 100 μl of sterilized PBS) were injected into the right and left dorsal flanks (n = 5), respectively. The tumor volumes were measured every week with a micrometer caliper. Tumor volumes were calculated using the Formula V=length × width^2^/2. After injection for 5 weeks, the mice were euthanized and the tumor samples were removed. All of the procedures were approved by the Institution Animal Care Committee of Shandong Provincial Hospital Affiliated to Shandong First Medical University (Approval No. 2021-774).

### 2.11 Statistical analysis

The SPSS 18.0 statistical analysis software (IBM Corp., Armonk, NY, USA) was used to analyze the experimental data. All data are presented as the mean ± standard deviation (SD). The Student’s t test or one-way ANOVA was used to evaluate the significant differences between groups. Associations between the expression levels of NUF2 and ERBB3 were analyzed by the Pearson’s correlation. *P* values < 0.05 were considered statistically significant.

## 3 Results

### 3.1 NUF2 is upregulated in EOC and predicts poor prognosis

**Figure 1:**
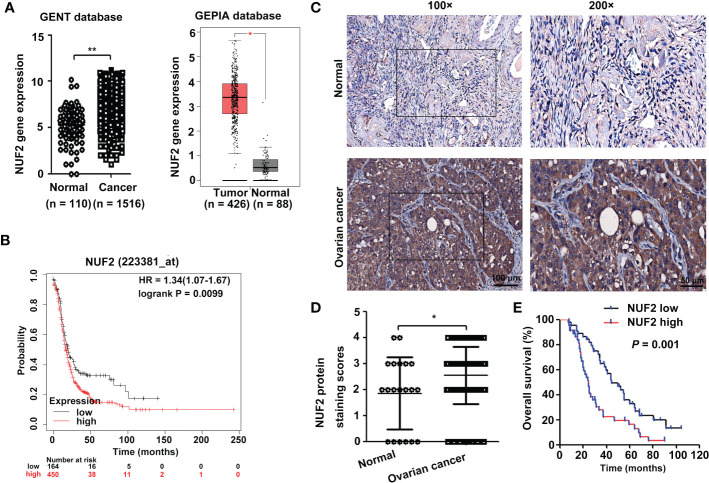
NUF2 expression is upregulated in EOC tissues. **(A)** NUF2 gene expression was determined in two databases (GENT2 and GEPIA). *P < 0.05, **P < 0.01. **(B)** Kaplan-Meier analysis based on Kaplan–Meier Plotter databases showed that patients with low NUF2 levels exhibited significantly better overall survival than patients with high NUF2 levels. **(C)** Representative images of IHC staining of NUF2 expression in EOC tissues and normal ovarian epithelial tissues are shown in the upper panel (original magnification, left × 100, right × 200). **(D)** NUF2 protein expression was significantly higher in 89 EOC tissues than in 20 normal ovarian epithelial tissues. *P < 0.05. **(E)** The High NUF2 expression was associated with poor EOC prognosis (*P* = 0.001).

The Gene Expression database of Normal and Tumor tissue 2 (GENT2) (https://gent2.appex.kr/gent2/) (19) and the Gene Expression Profiling Interactive Analysis (GEPIA) database (http://gepia.cancer-pku.cn/) (20) were used to determine the expression of NUF2 in EOC. We found that NUF2 gene expression was significantly upregulated in EOC (**Figure 1A**). To further verify the effect of NUF2 expression on the prognosis of EOC, we performed survival analysis using an online database. Data from the Kaplan–Meier Plotter database (http://www.kmplot.com) (21) showed that patients with higher NUF2 mRNA expression had worse overall survival than patients with lower NUF2 expression (**Figure 1B**). We subsequently analyzed the expression of NUF2 protein in 89 EOC tissues and 20 normal ovarian epithelial tissues. As shown in **Figures 1C, D**, NUF2 was found to be increased in EOC tissues compared to normal ovarian epithelial tissues. High NUF2 expression was associated with poor EOC prognosis (**Figure 1E**). In addition, we analyzed the association between the NUF2 expression and clinicopathological features in 89 EOC samples (**Table S3**). We found that high NUF2 expression was associated with FIGO stage (*P* = 0.007) and pathological grade (*P* = 0.015) (**Table 1**). Univariate analysis indicated that the FIGO stage and upregulated NUF2 expression were associated with overall survival (*P* = 0.026 and *P* = 0.043, respectively) (**Table 2**). Multivariate analysis showed that the FIGO stage and upregulated NUF2 expression were independent prognostic factors for overall survival (*P* = 0.008 and *P* = 0.003, respectively) (**Table 2**).

**Table 1:** Relationship between NUF2 expression and clinicopathological characteristics in 89 patients with EOC.

Characteristics	All cases	NUF2		*P* value
		Low expression	High expression	
Age (years)				0.169
< 50	47	20	27	
≥ 50	42	24	18	
Tumor diameter (cm)				0.597
< 10	44	23	21
≥10	45	21	24
FIGO stage				0.007**
I+II	52	32	20
III+IV	37	12	25
Pathological grade				0.015*
G1	39	25	14
G2+G3	50	19	31
Lymph node metastasis				0.069
Negative	77	41	36
Positive	12	3	9
Histological type				0.603
Serous	51	24	27
Other types	38	20	18

*Statistically significant (P < 0.05), **Statistically significant (P < 0.01).

**Table 2:** Cox proportional hazard models for prognostic factors.

	Univariate analysis		Multivariate analysis	
	HR(95% CI)	*P* value	HR(95% CI)	*P* value
Age (≥ 50 *vs.* < 50)	0.869 (0.530-1.425)	0.578		
Tumor diameter (≥10 *vs.* ≥10)	1.152 (0.691-1.922)	0.587		
FIGO stage (III+IV *vs.* I+II)	1.833 (1.074-3.130)	0.026*	1.948 (1.191-3.186)	0.008**
Pathological grade (G2+G3 *vs.* G1)	1.320 (0.773-2.255)	0.310		
Lymph node metastasis (positive *vs.* negative)	1.702 (0.818-3.541)	0.155		
Histological type (Other types *vs.* Serous)	0.972 (0.592-1.597)	0.911		
NUF2 expression (high vs. low)	1.778 (1.019-3.100)	0.043*	2.135 (1.305-3.494)	0.003**

*Statistically significant (P < 0.05), **Statistically significant (P < 0.01).

### 3.2 NUF2 downregulation inhibits EOC cell proliferation, migration and invasion *in vitro*

**Figure 2:**
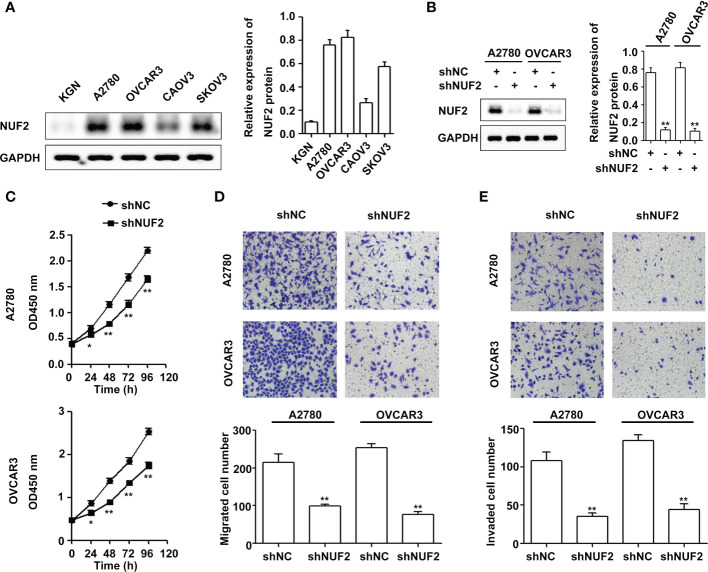
NUF2 downregulation inhibits EOC progression *in vitro*. **(A)** The protein levels of NUF2 were determined in four human EOC cell lines (A2780, OVCAR3, CAOV3 and SKOV3) and one human immortalized EOC cell line (KGN). **(B)** NUF2 expression in A2780 and OVCAR3 cells after transfection with shNC and shNUF2 was detected by Western blot. **P < 0.01 vs. the shNC group. **(C)** A CCK-8 assay was used to determine the viability of A2780 and OVCAR3 cells after transfection with shNC and shNUF2. *P < 0.05, **P < 0.01 vs. the shNC group. **(D, E)** Transwell assays were used to determine the migration and invasion of A2780 and OVCAR3 cells after transfection with shNC and shNUF2 (original magnification, × 200). **P < 0.01 vs. the shNC group.

To determine the role of NUF2 in EOC, we first analyzed the expression of NUF2 in four human EOC cell lines (A2780, OVCAR3, CAOV3 and SKOV3) and one human immortalized EOC cell line (KGN). We found that NUF2 expression in KGN was the lowest among the cell lines, and NUF2 expression was higher in the A2780 and OVCAR3 cell lines than in the CAOV3 and SKOV3 cell lines (**Figure 2A**). The A2780 and OVCAR3 cell lines were selected for the subsequent experiments. Then, we established stable A2780 and OVCAR3 cells with NUF2 silencing. As shown in **Figure 2B**, NUF2 shRNA significantly inhibited NUF2 protein levels in A2780 and OVCAR3 cells. To determine the effect of NUF2 on EOC cell proliferation, a CCK-8 assay was performed. Our results showed that NUF2 knockdown significantly repressed the proliferation capacity of A2780 and OVCAR3 cells when compared with those transfected with shNC (**Figure 2C**). Moreover, we observed that the migratory and invasive capacities of A2780 and OVCAR3 cells transfected with shNUF2 were significantly inhibited compared with those transfected with shNC (**Figures 2D, E**).

### 3.3 Global gene expression changes in OVCAR3 cells transfected with shNUF2 and shNC

**Figure 3:**
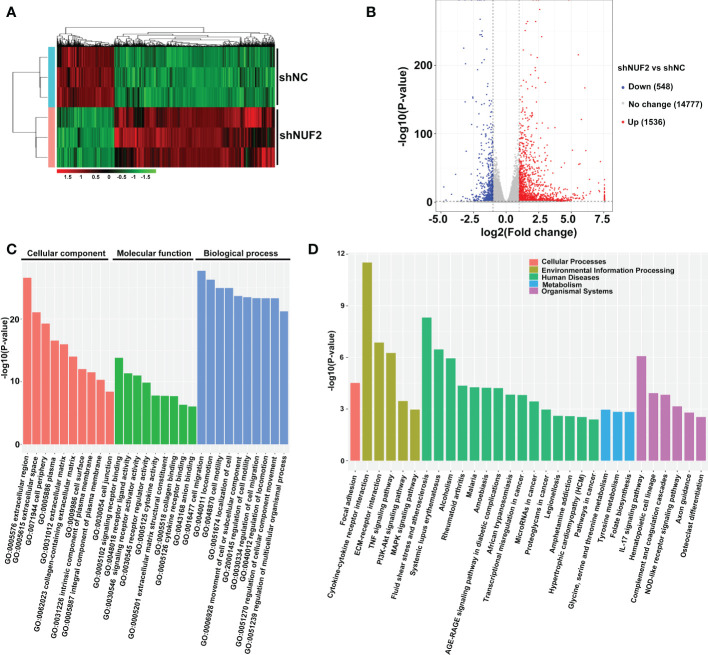
Global gene expression changes in OVCAR3 cells transfected with shNUF2 and shNC. **(A, B)** Heatmaps and volcano plots of the DEGs (FC, <-1-fold or >1-fold) in OVCAR3 cells infected with shNUF2 and shNC. **(C, D)** GO analysis and KEGG pathway enrichment of the DEGs in OVCAR3 cells infected with shNUF2 and shNC.

To investigate the underlying mechanism of NUF2 in regulating EOC progression, RNA-seq was performed to identify the signaling pathways influenced by NUF2. Gene expression heatmaps and volcano plots showed that a total of 548 genes were downregulated (FC, <-1-fold) and 1536 genes were upregulated (FC, >1-fold) (**Figures 3A, B**). RNA-seq data have been submitted to the GEO repository (series entry GSE213611)(https://www.ncbi.nlm.nih.gov/geo/query/acc.cgi?acc=GSE213611). To identify genes and pathways affected by NUF2, we performed GO and KEGG pathway enrichment. The top 10 GO terms, covering cellular component, molecular function, and biological process, are shown in **Figure 3C** and **Datasheet_1**. DEGs were obviously enriched in relevant terms, such as cell migration, cell motility, regulation of cell motility and regulation of cell migration (**Figure 3C**). As shown in **Figure 3D** and **Datasheet_2**, the KEGG results showed that the differentially expressed gene sets were significantly related to focal adhesion, the PI3K-AKT signaling pathway and the MAPK signaling pathway. These results verified the role of NUF2 in EOC cell migration and invasion, which were consistent with our observations.

### 3.4 NUF2 downregulation inhibits ERBB3 expression in EOC cells

**Figure 4:**
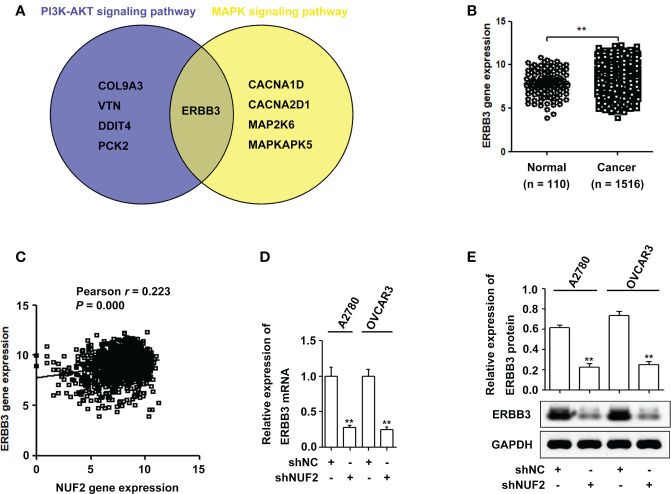
NUF2 downregulation inhibits ERBB3 expression in EOC cells. **(A)** A Venn diagram was used to represent common genes between the PI3K-AKT and MAPK signaling axes. **(B)** ERBB3 gene expression was significantly higher in EOC tissues than in the normal ovarian epithelial tissues. **P < 0.01. **(C)** The Pearson correlation analysis was used to explore the association between NUF2 and ERBB3 expression (*r* = 0.223, *P* = 0.000). **(D)** and **(E)** The mRNA and protein levels of ERBB3 were significantly reduced in NUF2 silenced cells when compared with A2780 and OVCAR3 cells transfected with shNC. **P < 0.01 vs. the shNC group.

Studies have shown that the PI3K/AKT and MAPK signaling pathways are essential for EOC progression (22, 23). We retrieved the downregulated gene sets from the PI3K/AKT and MAPK signaling pathways, and the results of the two gene sets were integrated by drawing a Venn diagram. As shown in **Figure 4A** and **Table S4**, ERBB3 was the overlapping gene in the PI3K/AKT and MAPK signaling pathways. Interestingly, ERBB3 is involved in the progression and metastasis of ovarian cancer (24). The GENT2 database showed that ERBB3 gene expression was significantly upregulated in EOC and was positively correlated with the expression of NUF2 (**Figures 4B, C**). Furthermore, the mRNA and protein levels of ERBB3 were significantly reduced in the A2780 and OVCAR3 cells transfected with shNUF2 compared with the shNC cells (**Figures 4D, E**). Based on literature reports and informatics analysis, we chose ERBB3 for further research.

### 3.5 ERBB3 downregulation inhibits EOC progression *via* the PI3K-AKT and MAPK signaling pathways

**Figure 5:**
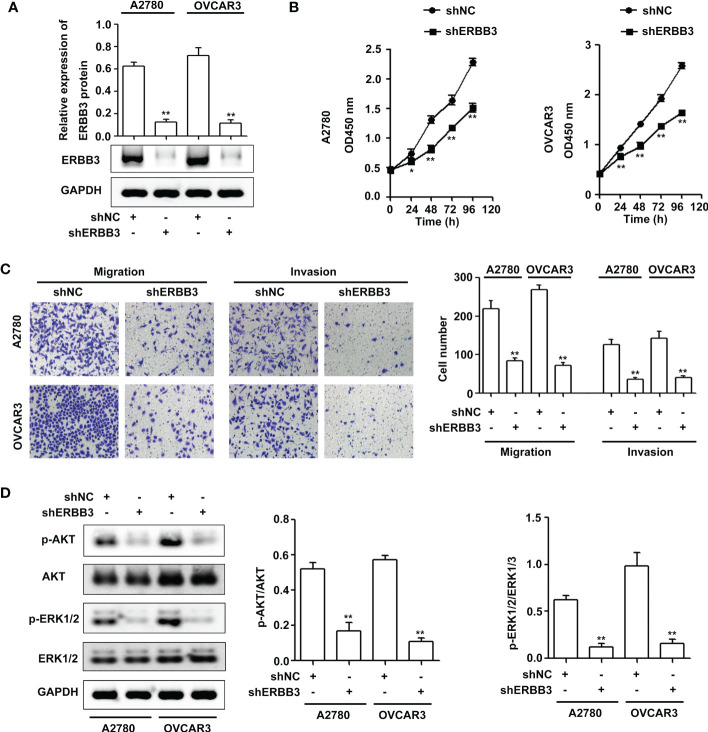
ERBB3 downregulation inhibits EOC progression *via* the PI3K-AKT and MAPK signaling axes. **(A)** NUF2 expression in A2780 and OVCAR3 cells after transfection with shNC and shERBB3 was detected by western blot. **P < 0.01 vs. the shNC group. **(B)** A CCK-8 assay was used to determine the viability of A2780 and OVCAR3 cells after transfection with shNC and shERBB3. *P < 0.05, **P < 0.01 vs. the shNC group. **(C)** Transwell assays were used to determine the migration and invasion of A2780 and OVCAR3 cells after transfection with shNC and shERBB3 (original magnification, × 200). **P < 0.01 vs. the shNC group. **(D)** Effects of ERBB3 silencing on the phosphorylation of AKT and ERK1/2. GAPDH was used as an internal control. **P < 0.01 vs. the shNC group.

To confirm the effect of ERBB3 on EOC progression, ERBB3 shRNA was transfected into A2780 and OVCAR3 cells. As shown in **Figure 5A**, ERBB3 protein levels were significantly reduced in the shERBB3-transfected cells compared with shNC-transfected cells. The CCK-8 assay showed that ERBB3 knockdown significantly suppressed the viability of A2780 and OVCAR3 cells (**Figure 5B**). In addition, the Transwell assay showed that ERBB3 knockdown significantly inhibited the migration and invasion of A2780 and OVCAR3 cells (**Figure 5C**). Western blot assays indicated that the levels of p-AKT and p-ERK1/2 were significantly downregulated by ERBB3 inhibition in the A2780 and OVCAR3 cells (**Figure 5D**).

### 3.6 NUF2 promotes EOC progression by the ERBB3-mediated PI3K-AKT and MAPK signaling axes

**Figure 6:**
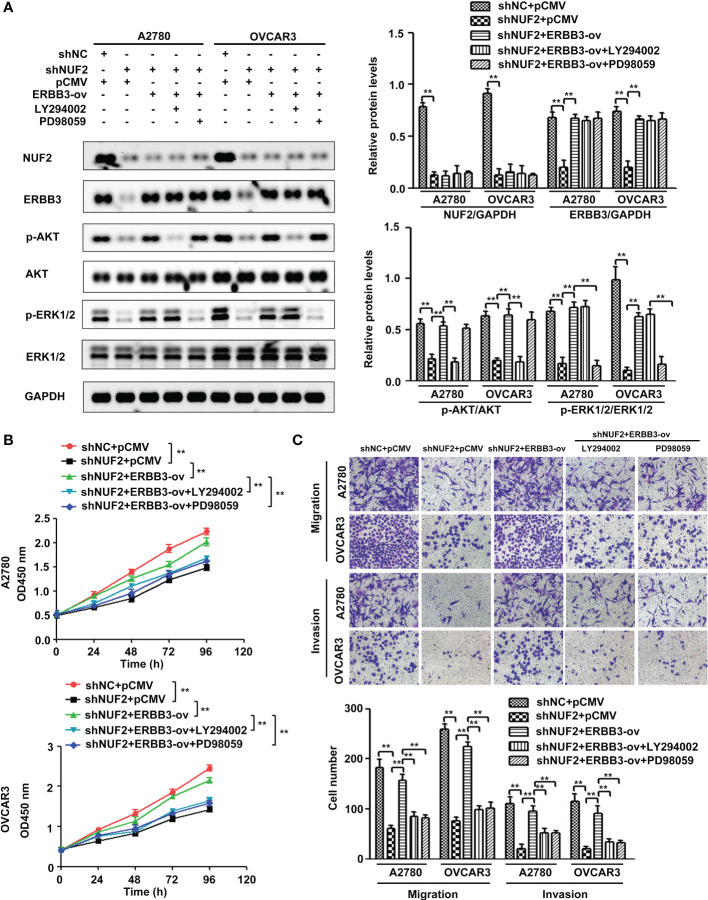
NUF2 promotes EOC progression by the ERBB3-mediated PI3K-AKT and MAPK signaling axes. **(A)** A2780 and OVCAR3 cells were treated with shNC+pCMV, shNUF2+pCMV, shNUF2+ERBB3-ov, shNUF2+ERBB3-ov+LY294002 (15 µM), and shNUF2+ERBB3-ov+PD98059 (10 µM). Western blotting was used to determine the protein expression of NUF2, ERBB3, p-AKT, AKT and p-ERK1/2, ERK1/2. **(B, C)** CCK-8 and transwell assays were used to determine the effects of ERBB3 restoration on the proliferative, migratory and invasive capacities of NUF2-downregulated cell lines. **P < 0.01. original magnification, × 200.

**Figure 7:**
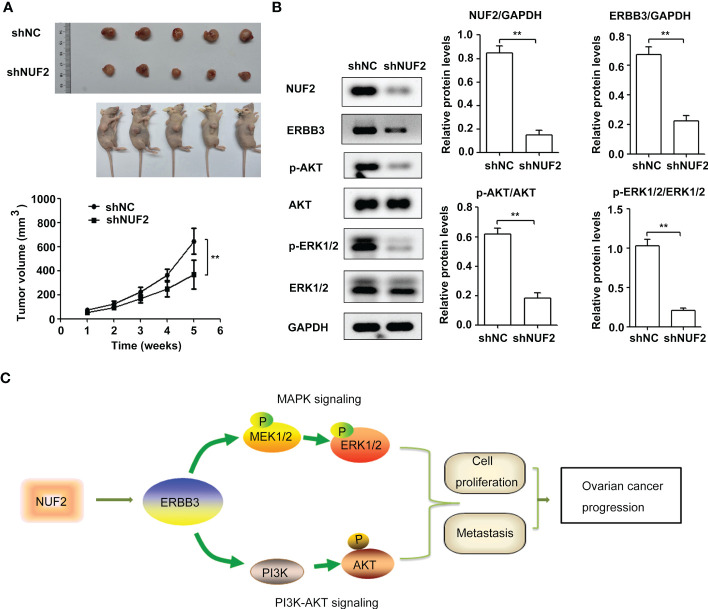
NUF2 knockdown repressed EOC tumorigenesis and inhibited ERBB3, p-AKT and p-ERK1/2 expression *in vivo*. **(A)** Representative photographs of mice and OVCAR3-shNUF2 and OVCAR3-shNC tumor samples 5 weeks after injection. Tumor volumes were measured at the indicated time points in all mice. **P < 0.01 vs. the shNC group. **(B)** Western blot assay was used to determine the expression levels of ERBB3, p-AKT and p-ERK1/2 proteins in tumors. **P < 0.01. **(C)** Schematic diagram summarizing the role and mechanism of NUF2 in promoting EOC progression. NUF2 promotes the PI3K-AKT and MAPK signaling axes mediated by ERBB3, thereby inducing the malignant behaviors in ovarian cancer cells.

Since ERBB3 expression was regulated by NUF2 in EOC cells, we further determined whether NUF2 promoted EOC progression by mediating ERBB3. ERBB3 expression plasmids together with shNUF2 plasmids were transfected into A2780 and OVCAR3 cells. As shown in **Figure 6A**, ERBB3 expression plasmids significantly reversed the inhibition of ERBB3 expression induced by shNUF2 in A2780 and OVCAR3 cells. CCK-8 and Transwell assays showed that the restoration of ERBB3 expression partially reversed the proliferative, migratory and invasive capacities of A2780 and OVCAR3 cells inhibited by NUF2 repression (**Figures 6B, C**). Western blot assays showed that the restoration of ERBB3 expression partially reversed the downregulation of p-AKT and p-ERK1/2 expression induced by NUF2 knockdown (**Figure 7A**). To determine whether ERBB3 regulates EOC progression by PI3K-AKT and MAPK signaling axes, two independent inhibitors of the PI3K (LY294002, inhibits AKT activity) and MAPK (PD98059, inhibits ERK activity) signaling pathways were used in A2780 and OVCAR3 cells transfected with ERBB3 expression plasmids together with shNUF2 plasmids. The levels of p-AKT and p-ERK1/2 induced by ERBB3 expression plasmids were decreased by LY294002 and PD98059 in A2780 and OVCAR3 cells transfected shNUF2, respectively (**Figure 6A**). In addition, cell proliferation, migration and invasion induced by ERBB3 expression plasmids were partially reversed by LY294002 and PD98059 in A2780 and OVCAR3 cells transfected with shNUF2 (**Figures 6B, C**).Thus, these results suggest that NUF2 activates the PI3K-AKT and MAPK signaling axes mediated by ERBB3, which regulates the malignant behaviors in EOC cells.

### 3.7 NUF2 downregulation inhibited EOC tumorigenesis and induced the suppression of the ERBB3 expression and the PI3K-AKT and MAPK signaling axes *in vivo*

To further confirm the biological functions of NUF2, we established a xenograft tumor model by inoculating OVCAR3 cells transfected with shNUF2 or shNC and monitored tumor size. The results showed that the tumor size of OVCAR3 cells transfected with shNUF2 was smaller than that of OVCAR3 cells transfected with shNC (**Figure 7A**). Western blot assays showed that ERBB3, p-AKT and p-ERK1/2 expression levels were significantly inhibited in the shNUF2 group when compared to the shNC group (**Figure 7B**). Thus, these results suggest that NUF2 activates the PI3K-AKT and MAPK signaling axes mediated by ERBB3, which regulates the malignant behaviors in EOC cells (**Figure 7C**).

## 4 Discussion

NUF2 is an essential component of the kinetochore-associated NDC80 complex that plays a regulatory role in chromosome segregation and spindle checkpoint activity in mitosis (6). Several studies have shown that NUF2 is upregulated in multiple cancers and is associated with poor prognosis (8–12). In the present study, we found that NUF2 mRNA and protein expression levels were higher in EOC tissues than in normal tissues (**Figures 1A, D**). Consistent with our results, one report also showed that NUF2 mRNA expression levels were significantly elevated in ovarian carcinoma tissues when compared with those in normal ovarian tissues (17). Moreover, our results showed that patients with low NUF2 expression levels exhibited a longer overall survival rate than patients with high NUF2 expression levels (**Figures 1B, E**). Univariate and Multivariate analyses indicated that upregulated NUF2 expression was associated with overall survival and was an independent prognostic factor for overall survival (**Table 2**). NUF2 was also aberrantly overexpressed in ovarian carcinoma cell lines (**Figure 2A**). Thus, NUF2 may be a novel prognostic biomarker for EOC.

Previous studies have indicated that NUF2 functions as an oncogene in different types of malignant tumors (9, 13–16). For instance, NUF2 depletion induces apoptosis and causes alterations in cell cycle distribution by inducing cell cycle arrest at the G0/G1 phase (9). Moreover, one report showed that NUF2 inhibition repressed cell viability and induced apoptosis in EOC cells (17). Consistent with these results, we also found that silencing NUF2 significantly inhibited cell proliferation *in vitro* (**Figure 2C**) and tumor growth *in vivo* (**Figure 7A**). Thus, NUF2 acts as an oncogene in EOC cells. Peritoneal dissemination is the main metastatic process of EOC, which generally leads to a sharp rise in the clinical stage and poor clinical prognosis (25). Thus, there is an urgent need to discover the mechanism of peritoneal dissemination to increase survival rates in ovarian cancer patients. Interestingly, NUF2 knockdown significantly inhibited pancreatic ductal adenocarcinoma cell migration and invasion (26). In this study, we demonstrate for the first time that NUF2 knockdown significantly reduced the migration and invasion in EOC cells (**Figures 2D, E**). Thus, NUF2 may be an ideal therapeutic target for EOC.

To elucidate the underlying mechanisms of EOC progression elicited by NUF2, we performed RNA-seq analysis to evaluate the DEGs in OVCAR3 cells after treatment with shNUF2 and shNC. As shown in **Figure 3C**, DEGs were obviously enriched in relevant terms, such as cell migration, cell motility, regulation of cell motility and regulation of cell migration. The KEGG results showed that the differentially expressed gene sets were significantly related to focal adhesion, the PI3K-AKT signaling pathway and the MAPK signaling pathway (**Figure 3D**). It has been reported that both the PI3K/AKT and MAPK signaling pathways are involved in EOC progression (27, 28). This may further explain the function of NUF2 in EOC cell migration and invasion. In addition, NUF2 is a key element of the Ndc80 kinetochore complex, which contributes to kinetochore–microtubule attachment and spindle assembly in mitosis (29). Interestingly, MAPK physically interacts with and regulates microtubule dynamics under certain unique circumstances such as meiosis (30). The microtubule-binding domain (MTBD) of the microtubule-associated protein 4 (MAP4) binds directly to the C2 domain of the p110α catalytic subunit and controls the interaction of PI3Kα with activated receptors at endosomal compartments along microtubules (31). Thus, increased NUF2 in EOC and other cancers may directly or indirectly regulate the PI3K-Akt and MAPK signaling because of NUF2 link with microtubules. Among the DEGs, ERBB3 overlapped in the PI3K/AKT and MAPK signaling pathways (**Figure 4A**), which is involved in the progression and metastasis of ovarian cancer and many other cancers (32–37). It is reported that ERBB3, the only member of the ErbB family incorporating multiple PI3k binding sites, is a major recruiter of PI3K (38). When ERBB3 binds to the regulatory p85 subunit of PI3K, the p110α catalytic subunit of PI3K is recruited, and AKT is then activated by PDK1 and mTORC2 (39). In the ERBB3 C terminus, there is a specific residue (Tyr1325), which contributes to the binding of SHC. And the mutagenesis of Tyr1325 abolished the interaction of ERBB3 with SHC, which could not effectively activate the Ras/MAPK signaling pathway (40). Moreover, we showed ERBB3 gene expression was significantly upregulated in EOC and was positively correlated with NUF2 expression (**Figures 4B, C**). Similar to the function of NUF2, ERBB3 knockdown significantly inhibited EOC cell proliferation, migration and invasion (**Figures 5B, C**). In addition, the levels of p-AKT and p-ERK1/2 were significantly downregulated by ERBB3 inhibition in EOC cells (**Figure 5D**). Thus, it is reasonable to speculate that NUF2 promotes EOC progression by ERBB3-mediated activation of the PI3K/AKT and MAPK signaling pathways. As expected, we found that ERBB3 restoration reversed the inhibition of proliferation, migration and invasion of EOC cells caused by NUF2 knockdown (**Figure 6**). ERBB3 restoration partially reversed the downregulation of p-AKT and p-ERK1/2 expression induced by NUF2 knockdown (**Figure 6A**). In addition, two independent inhibitors of the PI3K (LY294002, inhibits AKT activity) and MAPK (PD98059, inhibits ERK activity) signaling pathways were used in A2780 and OVCAR3 cells transfected with ERBB3 expression plasmids together with shNUF2 plasmids. We found that cell proliferation, migration and invasion induced by ERBB3 expression plasmids were partially reversed by LY294002 and PD98059 in A2780 and OVCAR3 cells transfected with shNUF2 (**Figure 6B, C**). These results indicated that NUF2 promoted EOC progression by inducing activation of the PI3K/AKT and MAPK signaling pathways *via* regulating ERBB3.

In summary, our study is the first to elucidate the role and mechanism of NUF2 in EOC cell migration and invasion *via* ERBB3-mediated activation of the PI3K/AKT and MAPK signaling pathways. However, the mechanism of how NUF2 affects ERBB3 expression deserves further exploration. A recent report showed that NUF2 promotes clear cell renal cell carcinoma progression through epigenetic activation of high-mobility group AT-hook 2 (HMGA2) transcription by suppressing lysine demethylase 2A (KDM2A) expression and affecting its occupancy on the HMGA2 promoter region to regulate histone H3 lysine 36 di-methylation (H3K36me2) modification (41). Thus, does NUF2 affect the promoter activity of ERBB3 gene? These mechanisms will be our future research direction.

## Data availability statement

The datasets presented in this study can be found in online repositories. The names of the repository/repositories and accession number(s) can be found in the article/**Supplementary Material**.

## Ethics statement

The studies involving human participants were reviewed and approved by Shandong Provincial Hospital Affiliated to Shandong First Medical University. The patients/participants provided their written informed consent to participate in this study. The animal study was reviewed and approved by Shandong Provincial Hospital Affiliated to Shandong First Medical University.

## Author contributions

RL and YM conceived and designed the study. RL, XS, and YZ performed experiments. RL and YM analyzed the data. RL wrote the manuscript. All authors contributed to the article and approved the submitted version.
