# Supplementary material for: NUF2 overexpression contributes to epithelial ovarian cancer progression via ERBB3-mediated PI3K-AKT and MAPK signaling axes
Source: Front Oncol. 2022 Dec 21;12:1057198. doi: 10.3389/fonc.2022.1057198 (PMC9811817; doi:10.3389/fonc.2022.1057198)
Supplement: Supplementary file 1 [file Table_1.docx]

**Table S1.** The sequences of shRNAs

| Name | Sequence | The region bound |
| --- | --- | --- |
| shNUF2 | 5′-CCGGCTTCTTACCATTCAGCAATTTCTCGAG AAATTGCTGAATGGTAAGAAGTTTTTTG-3′ | CDS |
| shERBB3 | 5′-CCGGAATTCTCT ACTCTACCATTGCTCGAGCAATGGTAGAGTAGAGAATTTTTTG-3′ | CDS |
| shNC | 5′- CCGGTTCTCCGAACGTGTCACGTTTCAAGAGAACGTGACACGTTCGGAGAATTTTTTG-3′ | / |
