# Supplementary material for: NUF2 overexpression contributes to epithelial ovarian cancer progression via ERBB3-mediated PI3K-AKT and MAPK signaling axes
Source: Front Oncol. 2022 Dec 21;12:1057198. doi: 10.3389/fonc.2022.1057198 (PMC9811817; doi:10.3389/fonc.2022.1057198)
Supplement: Supplementary file 2 [file Table_2.docx]

**Table S2.** The PCR primers

| Gene name | Sequence |
| --- | --- |
| ERBB3 sense | 5’-GCCAATGAGTTCACCAGGAT-3’ |
| ERBB3 antisense | 5’-ACGTGGCCGATTAAGTGTTC-3’ |
| GAPDH sense | 5’- GGTGAAGGTCGGAGTCAACG-3’ |
| GAPDH antisense | 5’-CAAAGTTGTCATGGATGHACC-3’ |
