# Supplementary material for: NUF2 overexpression contributes to epithelial ovarian cancer progression via ERBB3-mediated PI3K-AKT and MAPK signaling axes
Source: Front Oncol. 2022 Dec 21;12:1057198. doi: 10.3389/fonc.2022.1057198 (PMC9811817; doi:10.3389/fonc.2022.1057198)
Supplement: Supplementary file 3 [file Table_3.docx]

**Table S3.** **The stages and pathological types of 89 epithelial ovarian cancers collected in this study**

| Number | FIGO stage | Pathological grade |
| --- | --- | --- |
| 1 | III | G2 |
| 2 | II | G1 |
| 3 | II | G2 |
| 4 | II | G1 |
| 5 | II | G2 |
| 6 | III | G3 |
| 7 | III | G2 |
| 8 | I | G1 |
| 9 | I | G1 |
| 10 | III | G3 |
| 11 | I | G2 |
| 12 | II | G3 |
| 13 | III | G2 |
| 14 | I | G1 |
| 15 | III | G3 |
| 16 | I | G1 |
| 17 | II | G2 |
| 18 | II | G2 |
| 19 | III | G1 |
| 20 | III | G2 |
| 21 | I | G1 |
| 22 | III | G1 |
| 23 | II | G2 |
| 24 | IV | G2 |
| 25 | I | G1 |
| 26 | III | G2 |
| 27 | III | G2 |
| 28 | I | G1 |
| 29 | II | G2 |
| 30 | III | G3 |
| 31 | II | G2 |
| 32 | III | G3 |
| 33 | II | G2 |
| 34 | II | G2 |
| 35 | III | G1 |
| 36 | III | G1 |
| 37 | III | G3 |
| 38 | II | G2 |
| 39 | II | G1 |
| 40 | III | G2 |
| 41 | II | G1 |
| 42 | III | G2 |
| 43 | II | G3 |
| 44 | II | G1 |
| 45 | III | G3 |
| 46 | II | G1 |
| 47 | III | G2 |
| 48 | II | G2 |
| 49 | III | G1 |
| 50 | III | G2 |
| 51 | II | G1 |
| 52 | II | G1 |
| 53 | II | G3 |
| 54 | III | G1 |
| 55 | II | G1 |
| 56 | III | G2 |
| 57 | II | G1 |
| 58 | III | G2 |
| 59 | III | G3 |
| 60 | II | G1 |
| 61 | III | G3 |
| 62 | II | G1 |
| 63 | I | G2 |
| 64 | III | G1 |
| 65 | III | G2 |
| 66 | III | G1 |
| 67 | I | G1 |
| 68 | I | G1 |
| 69 | II | G2 |
| 70 | II | G1 |
| 71 | II | G1 |
| 72 | II | G2 |
| 73 | III | G2 |
| 74 | III | G2 |
| 75 | II | G2 |
| 76 | III | G1 |
| 77 | II | G1 |
| 78 | II | G3 |
| 79 | III | G3 |
| 80 | II | G2 |
| 81 | III | G2 |
| 82 | II | G1 |
| 83 | II | G1 |
| 84 | II | G1 |
| 85 | III | G2 |
| 86 | I | G1 |
| 87 | II | G2 |
| 88 | II | G1 |
| 89 | II | G1 |
