# Supplementary material for: NUF2 overexpression contributes to epithelial ovarian cancer progression via ERBB3-mediated PI3K-AKT and MAPK signaling axes
Source: Front Oncol. 2022 Dec 21;12:1057198. doi: 10.3389/fonc.2022.1057198 (PMC9811817; doi:10.3389/fonc.2022.1057198)
Supplement: Supplementary file 4 [file Table_4.doc]

**Table S4. PI3K-Akt signaling pathway and MAPK signaling pathway**

| ID | pathway | *P*-value | Down_gene |
| --- | --- | --- | --- |
| hsa04151 | PI3K-Akt signaling pathway | 0.0003 | ERBB3, COL9A3, VTN, DDIT4, PCK2 |
| hsa04010 | MAPK signaling pathway | 0.0010 | ERBB3, CACNA1D, CACNA2D1, MAP2K6, MAPKAPK5 |
