# Supplementary figures and images for: NUF2 overexpression contributes to epithelial ovarian cancer progression via ERBB3-mediated PI3K-AKT and MAPK signaling axes
Source: Front Oncol. 2022 Dec 21;12:1057198. doi: 10.3389/fonc.2022.1057198 (PMC9811817; doi:10.3389/fonc.2022.1057198)

Figure 2

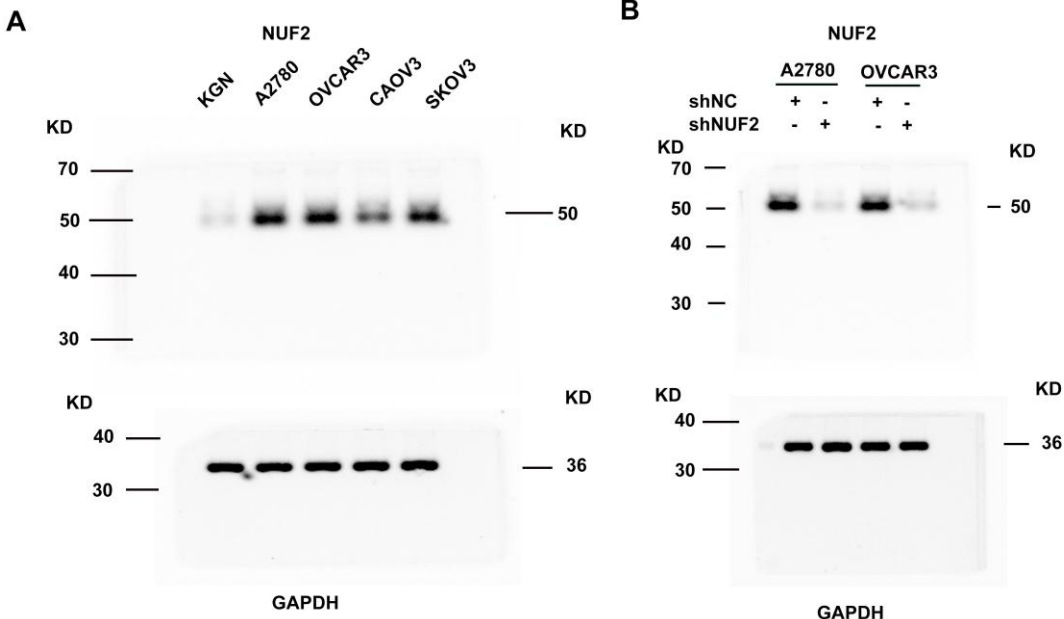

Figure 4E

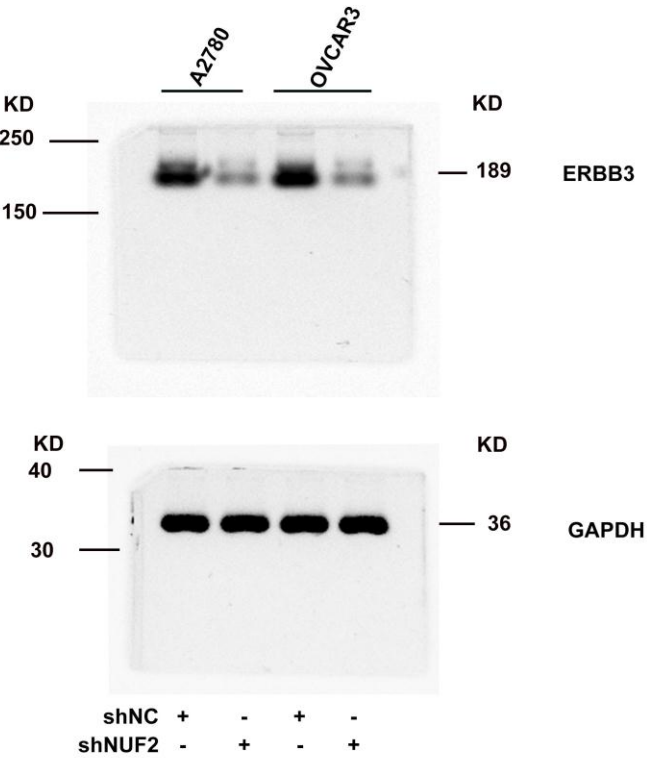

Figure 5

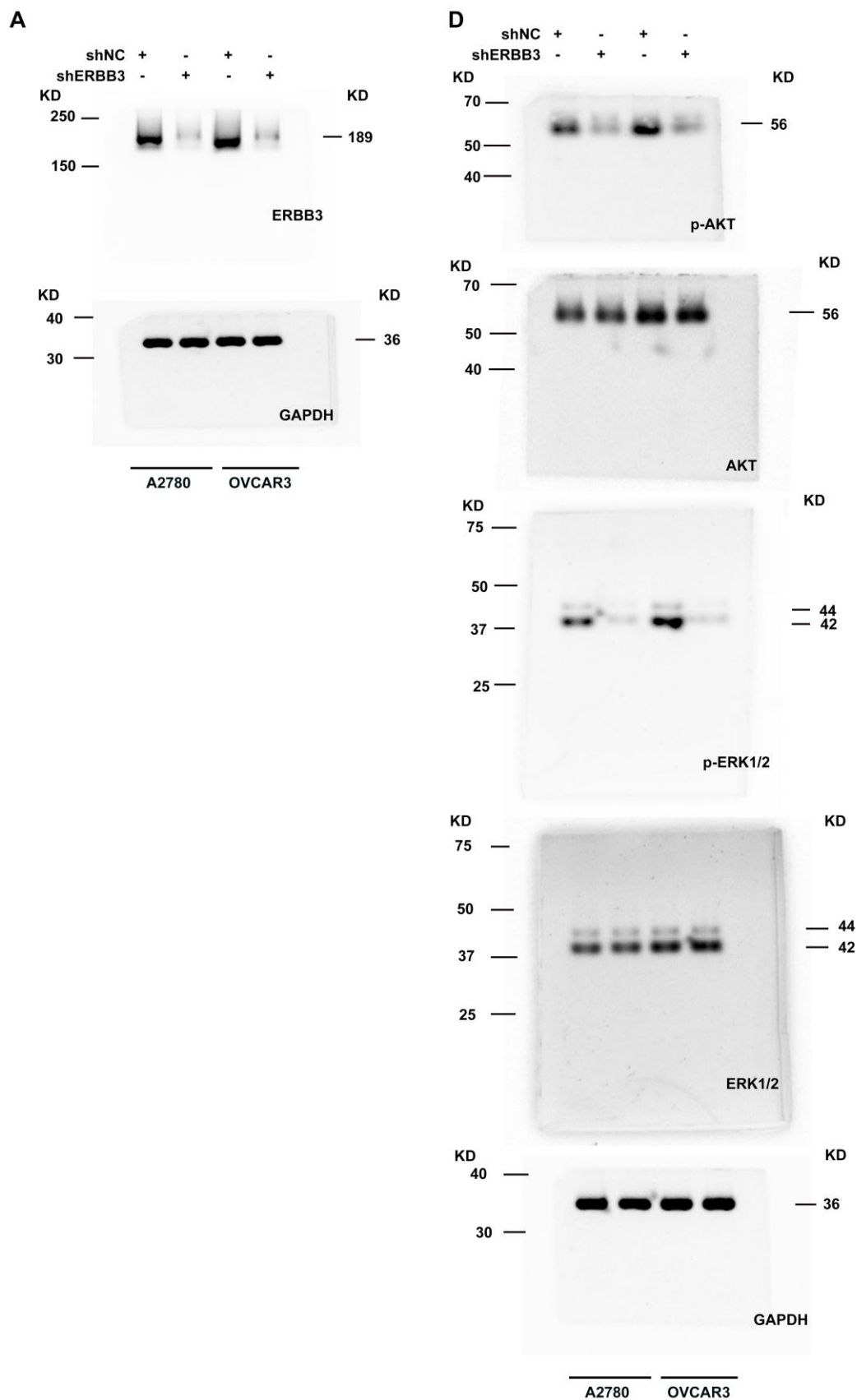

Figure 6A

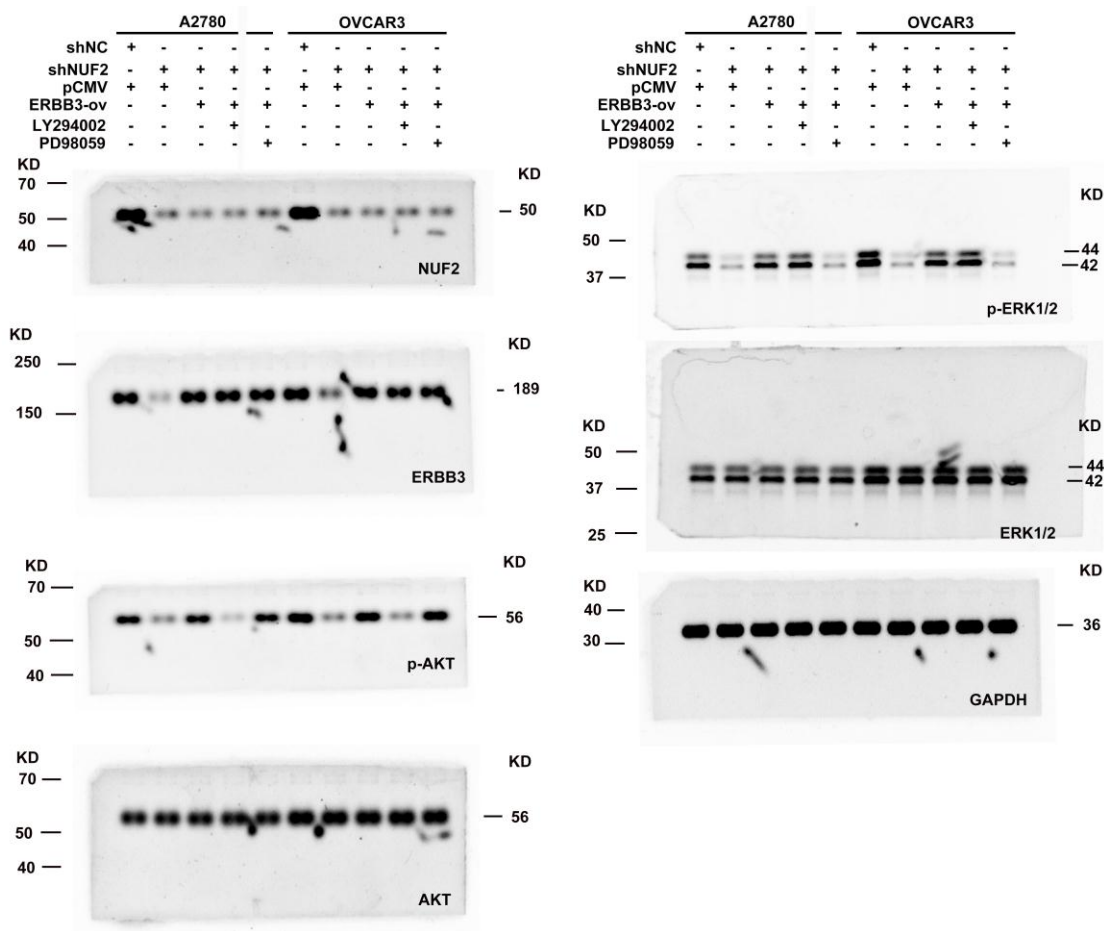

Figure 7B

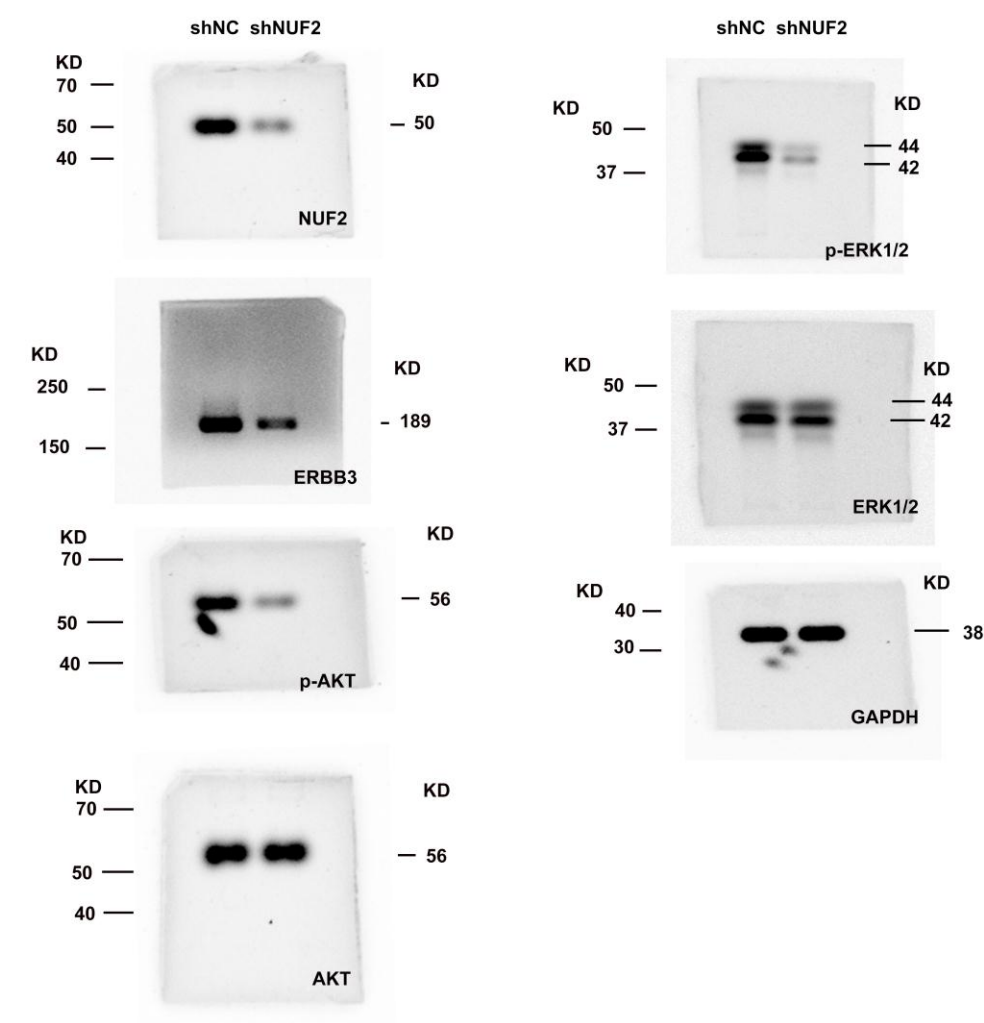

Supplement: Supplementary file 7 [file DataSheet_3.pdf]
